# Blow-in fracture of the orbital roof presenting as a case of non-resolving choroidal effusion

**DOI:** 10.4103/0301-4738.64131

**Published:** 2010

**Authors:** Bipasha Mukherjee, Muna Bhende

**Affiliations:** Department of Orbit, Oculoplasty & Trauma, Sankara Nethralaya, Chennai, India; 1 Vitreo-retina, Sankara Nethralaya, Chennai, India

**Keywords:** Blow-in fracture, choroidal effusion, ultrasonography

## Abstract

A 34-year-old male patient was referred to us as a case of non-resolving suprachoroidal hemorrhage. History revealed decrease in right eye vision following trauma to forehead. B scan ultrasonography (USG) of the right eye showed a high-reflective structure indenting the globe. It turned out to be an inferiorly displaced fracture fragment from the orbital roof on computerized tomography (CT) scan. The choroidal elevation disappeared after open reduction of the fracture fragment and patient had good recovery of vision. USG and CT scan were helpful in the diagnosis and management of this case.

There can be various reasons behind decrease in vision following trauma to the globe or adnexa. Here we present a unique case scenario wherein an undetected orbital fracture fragment caused choroidal elevation and decreased vision.

## Case Report

**Figure 1:**
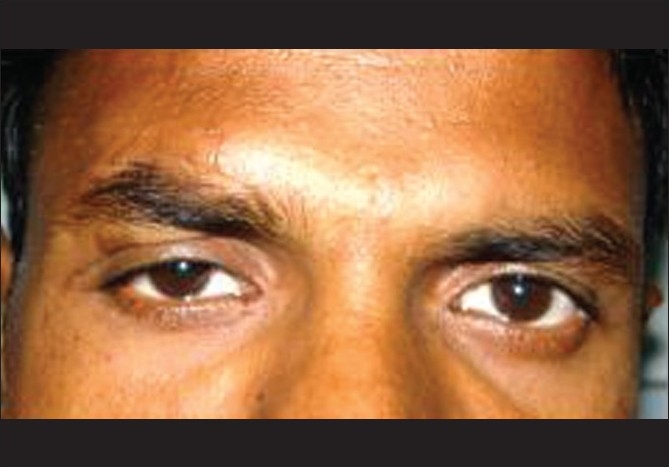
Clinical photograph of patient showing OD mild ptosis

A 34-year-old Indian male, a farmer by profession, reported to our institute with history of decrease in vision in the right eye since one month. He gave history of fall from his bicycle, leading to laceration on right forehead, which was sutured locally. He was diagnosed to have right suprachoroidal hemorrhage in the right eye and was treated with subtenon injections of triamcinolone acetate. He was referred to our institute as his symptoms did not improve. On examination, his vision in the right eye was 20/60; J4 and that in the left eye was 20/20; J1. Right eye showed mild ptosis with restricted elevation [Fig. 1]. The pupils of both the eyes were normal. Left eye anterior and posterior segment examination was within normal limits. Applanation tension was 7 and 9 mmHg respectively.

**Figure 2:**
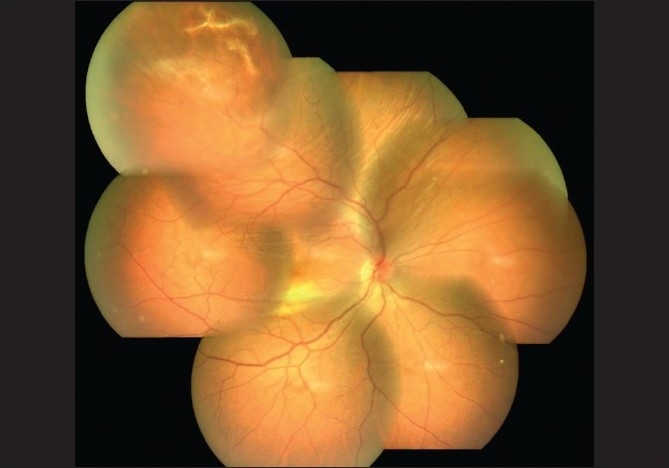
OD fundus montage photograph: showing elevation of the choroid in superotemporal quadrant

Fundus examination of the right eye showed a normal disc; internal limiting membrane (ILM) striae at the macula and a choroidal elevation in the superotemporal quadrant (STQ) with retinal pigment epithelial (RPE) changes and subretinal hemorrhages in the area [Fig. 2].

**Figure 3:**
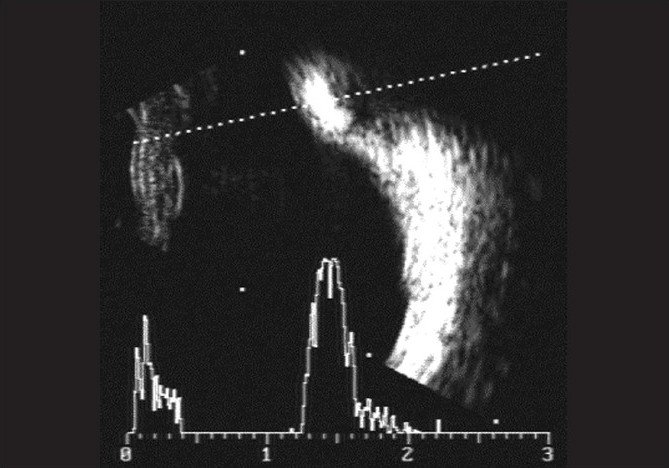
RE USG: Showing a high reflective lesion indenting the supero-temporal part of the globe

B scan ultrasonography (USG) showed a clear vitreous cavity with attached retina and normal-appearing choroid. Globe wall indentation was noted in the STQ, apparently caused by a linear intensely high-reflective structure causing significant orbital shadowing. It was suspected to be an inferiorly displaced fractured bone fragment [Fig. 3].

**Figure 4:**
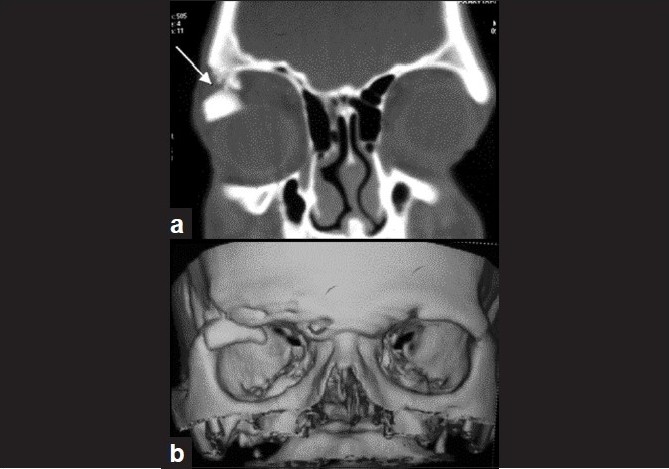
(a) CT scan (coronal view): RE roof fracture with inferior and medial displacement of fracture fragment (arrow) causing indentation of the right globe. (b) 3D reconstruction

The patient was examined in the department of orbit and trauma where on palpation the superior orbital rim of the right eye showed a discontinuity with minimal displacement of the globe inferiorly. Upper lid lag was noted. Periocular sensations were grossly normal. There was no proptosis/ enophthalmos. Computerized tomography (CT) scan (2-mm plain axial spiral followed by 3-mm coronal and three-dimensional reconstruction) was ordered, which revealed a comminuted fracture of the superior orbital rim of the right eye with inferior and medial displacement of the fragment into the orbit. The fractured fragment was obliquely placed and abutting the superior ocular surface and superior rectus/levator palpabrae superioris complex. The right globe was displaced inferiorly [Fig. 4].

**Figure 5:**
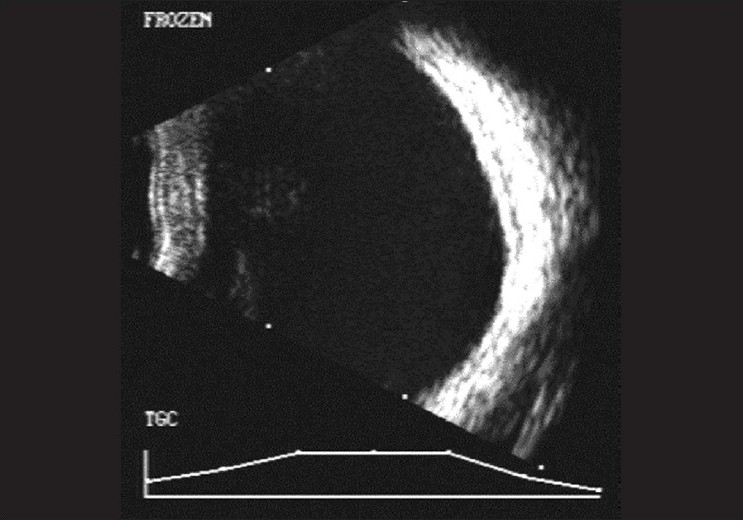
Post-operative USG: showing normal globe contour

The patient was taken up for open reduction of fracture fragment under general anesthesia. Superior lid crease was marked and incision was given. Dissection was carried out till the superior orbital rim, where the periosteum was incised. The fracture fragment was identified and reduced. It was drilled and sutured in position with non-absorbable sutures. The postoperative period was uneventful. He was reviewed in the retina clinic five days after surgery when the choroidal elevation appeared decreased, repeat USG showed no evidence of globe indention [Fig. 5].

On last follow-up, six months after surgery, patient's vision was 20/30; J2 in the right eye and 20/20; J1 in the left eye. Extraocular motility (EOM) was full. There was complete resolution of right eye ptosis with comparable lid heights in both eyes. Right eye fundus examination showed RPE changes in the area of choroidal folds and RPE atrophic areas in STQ. No choroidal elevation was seen.

## Discussion

The differential diagnosis of choroidal elevation includes serous or hemorrhagic detachments, intraocular tumors, ocular inflammations such as scleritis and granulomas or orbital masses causing globe indentation.[1] Ours is an unusual case where a bone fragment from the orbital roof got displaced causing a similar appearance and resulted in a diagnostic dilemma.

"Blow-in" fractures of the orbital roof result from a significant direct blunt force applied to the supraorbital region of the frontal bone. This results in transmission of energy to the thin orbital plate of this bone and displacement of bone fragments downward into the superior orbit.[2] B-scan USG was able to detect the displaced bone fragment causing globe wall indentation and apparent choroidal elevation. High-resolution CT with multiplanar reformation and three-dimensional display proved very useful in identifying and characterizing the bone and soft tissue abnormalities found in our patient. Prompt surgical intervention to reduce the fractured segment helped us to reverse the globe indentation.

In conclusion, we report an unusual case of undetected orbital roof blow-in fracture presenting as a non-resolving choroidal detachment. This case also emphasizes the importance of opportune imaging techniques in cases with unusual presentations not responsive to conventional treatment.
